# Randomized Controlled Trial of Active Physician-Based Surveillance of Foodborne Illness

**DOI:** 10.3201/eid0801.010356

**Published:** 2002-01

**Authors:** Zhong Dong, Mark J. Ferson, Peter Yankos, Valerie Delpech, Richard Hurst

**Affiliations:** * School of Community Medicine, University of New South Wales, Kensington, New South Wales, Australia; † South Eastern Sydney Public Health Unit, Zetland, New South Wales, Australia; ‡ St George Division of General Practice, Carlton, New South Wales, Australia

**To the Editor:** In New South Wales, Australia, physicians are obliged to report to public health authorities instances of foodborne illness in two or more cases related to a common source. This reporting of cases on a clinical basis complements laboratory-based surveillance of foodborne illness and is an essential form of surveillance in situations in which clinical samples may not be collected or in which specific diagnostic testing is not routinely available. Although cases of foodborne illness are increasing, substantial underreporting to health authorities is believed likely (*1*,*2*). However, reporting of foodborne illness and investigation of identified outbreaks are important forms of health protection (*1*–*4*).

In a pilot study, we examined whether notification of single (rather than multiple) cases, active surveillance, or both would improve the reporting of foodborne illness by family physicians and thus its detection in the community.

St. George Division of General Practice, one of four networks of family physicians located in the southeastern quadrant of Sydney within the jurisdiction of the South Eastern Sydney Public Health Unit, offered to participate in the study. Passive surveillance consisted of writing to all 329 members of the St. George Division asking them to report any single case of foodborne illness on a purpose-designed form that could be faxed to the Public Health Unit. Reports remained unidentifiable unless the patient gave the physician consent for Public Health Unit follow-up. The active surveillance group comprised 34 randomly selected St. George Division members who, in addition to being sent the written information, were contacted by telephone once every 3 weeks.

Over the 12-week study period from August to November 1999, St. George Division physicians made 39 reports , 31 (79%) by facsimile and 8 by mail; in contrast, no reports of foodborne illlness were received from the other 900 family physicians practicing in southeastern Sydney. Of the 39 notifications, 26 were received from 12 (35%) of 34 active surveillance physicians and 13 from 8 (2.7%) of the remaining 295 physicians (odds ratio 19.6 [95% confidence intervals 6.6-59]).

Consent was given for the Public Health Unit's food inspectors to follow up 13 cases, 6 of which represented multiple associated cases with possible public health implications. In one family, three members had pain, altered temperature sensation, and lower limb weakness 4 to 36 hours after eating portions of flowery cod; they were diagnosed as suffering from ciguatera poisoning. This potentially serious condition is caused by consumption of heat-stable ciguatoxin concentrated in the tissues of certain types of reef fish that have ingested toxin-producing plankton. Ciguatera poisoning has wide global distribution, including the Indo-Pacific and Caribbean regions (*5*); it has important public health implications because of its frequency and severity, and the fact that prompt recognition and treatment can lead to a good clinical outcome (*5*–*7*)

Better ascertainment of foodborne illness is required to improve food safety in Australia, including removing suspect foods from circulation (*1*,*3*). We found that passive surveillance of single cases increased the reporting of suspected foodborne illness by family physicians, while active surveillance based on telephone contacts elicited notification of clusters of associated cases, one of which required prompt public health action. At the least, this pilot suggests vast underreporting of food poisoning and that different strategies are available to improve reporting. A large-scale study would be required to determine the feasibility, acceptability, and value to public health of this form of enhanced surveillance.
